# Physical ACTivity in Survivorship (PACTS): study protocol for a randomized controlled trial evaluating a goal-directed therapeutic exercise program in pediatric posterior fossa brain tumor survivors

**DOI:** 10.1186/s12887-021-02566-7

**Published:** 2021-03-01

**Authors:** Brooke E. Kohler, Emmah Baque, Carolina X. Sandler, Denise S. K. Brookes, Caroline O. Terranova, Matthew Rixon, Tim Hassall, Stewart G. Trost

**Affiliations:** 1 Institute of Health and Biomedical Innovation at the Queensland Centre for Children’s Health Research, Queensland University of Technology (QUT), Brisbane, Queensland Australia; 2 School of Exercise and Nutrition Science, Queensland University of Technology (QUT), Brisbane, Queensland Australia; 3 School of Allied Health Sciences, Griffith University, Gold Coast, Queensland Australia; 4 UNSW Fatigue Research Program, Kirby Institute, University of New South Wales, Sydney, NSW Australia; 5 School of Clinical Sciences, Queensland University of Technology (QUT), Brisbane, Queensland Australia; 6 Queensland Children’s Hospital, Brisbane, Queensland Australia

**Keywords:** Oncology, Children, Physical activity, Cardiorespiratory fitness, Goal attainment, Quality of life

## Abstract

**Background:**

Posterior fossa brain tumors (PFBT) are the most common solid tumor in children. Recent increases in survival rates are encouraging; however, survivors may experience a plethora of disease- and treatment-related complications that can persist into adulthood. Therapeutic exercise interventions have been shown to improve quality of survivorship in other pediatric cancer diagnoses. There is also evidence that goal-directed interventions are effective at improving motor activities, function, and self-care in children with complex health conditions. Yet, there is currently no evidence on the efficacy of goal-directed therapeutic exercise in pediatric PFBT survivors. The Physical ACTivity in Survivorship (PACTS) study aims to investigate the effects of a novel goal-directed therapeutic exercise program on cardiorespiratory fitness and physical activity-related goal attainment in pediatric survivors of PFBT.

**Method:**

PFBT survivors, aged five to 17 years, who underwent surgery at least 12 months earlier and completed radiation therapy and/or chemotherapy at least 6 months prior will be recruited from the Queensland Children’s Hospital (Brisbane, Australia) (target *n* = 48). Following baseline assessment, participants are randomized into either the intervention or usual care group. The intervention group will receive weekly individualized, goal-directed exercise therapy delivered face-to-face for 12 weeks, along with an accompanying home-based program (three sessions per week). Outcomes will be assessed at baseline, immediately post-intervention, and at 6- and 12-months post-intervention. The primary outcomes are cardiorespiratory fitness (Peak VO_2_) and physical activity-related goal attainment. Secondary outcomes are cardiorespiratory endurance, high-level mobility skills, functional muscle strength, habitual physical activity, gait, balance, quality of life, fatigue, participation, perceived movement skill competence and parameters of body composition.

**Discussion:**

PACTS is the first study to investigate the efficacy of goal-directed therapeutic exercise in children with PFBT and provide evidence needed to inform clinical practice recommendations for managing quality of survivorship in PFBT survivors.

**Trial registration:**

ACTRN12619000841178.

**Supplementary Information:**

The online version contains supplementary material available at 10.1186/s12887-021-02566-7.

## Background

Posterior fossa brain tumors (PFBT) account for 25% of all pediatric cancer diagnoses and are the most common solid tumor in children [1, 2]. This cluster of diagnoses include medulloblastoma, ependymoma, cerebellar astrocytoma and brain stem glioma, with the former two showing increasing incidence rates over the last three decades [3, 4]. Advancements in medical management have led to significant improvements in survival, with five-year survival rates increasing by 5–10, to 70% over the last 30 years (United States and Australia) [2, 4]. The improved survival rates of PFBTs presents an ongoing challenge of how to maximize quality of survivorship and effectively manage the short- and long-term complications acquired from the disease and treatment [3]. For the purpose of this study, a ‘survivor’ is regarded as any patient who has completed treatment with curative intent.

The anatomical location of PFBTs, with or without administration of cranial radiation therapy, negatively impact the cerebellum and adjacent areas [5]. Consequently, pediatric survivors of PFBTs may experience several late effects following treatment, including motor, neurological, cognitive, and psychosocial complications [5–9]. Compared to typically-developing peers, childhood PFBT survivors are less able to participate in physical activity, maintain schoolwork, engage in conversations with family and peers, and participate in other age-appropriate activities such as learning to drive or household chores [10, 11]. Moreover, PFBT survivors report significantly poorer quality of life compared to healthy controls and other types of cancers [12, 13]. Fatigue is frequently reported in childhood cancer survivors, with nearly half experiencing persistent fatigue symptoms months to years after completion of treatment [14]. Fatigue may impact children’s meaningful engagement in school, recreation and home activities, through poor concentration, decreased attention, memory loss and activity intolerance [14, 15]. Mechanisms that underpin the activity limitations and participation restrictions associated with a PFBT and its treatment persist through many years of survivorship and increase an individual’s risk for disabling chronic health conditions. In adulthood, PFBT survivors exhibit higher rates of cardiovascular disease, obesity, diabetes and osteoporosis than the general population [16–19].

Pediatric PFBT survivors exhibit poor cardiorespiratory fitness, performing well below their age-matched peers and equivalent to children with congenital heart disease [6, 20]. Following treatment, PFBT survivors also demonstrate significant deficits in lower limb strength, balance, and motor proficiency [6, 8, 21, 22]. These physical impairments have been shown to persist into adulthood, with young adult survivors of childhood brain tumors demonstrating similar muscle strength to persons aged over 60 years [7, 23]. Survivors of childhood cancer engage in less physical activity than their typically-developing age-matched peers; and brain tumor survivors are less likely to meet physical activity guidelines than children with other cancer diagnoses [24–26]. Lifestyle factors such as physical inactivity are associated with increased risk of chronic disease, and regular participation in physical activity has been shown to be both preventative and therapeutic [27].

Intervention studies conducted in childhood cancer survivors indicate that therapeutic exercise is safe and feasible; however, studies conducted to date have focused predominantly on children with blood cancers (e.g., acute lymphoblastic leukemia) [28]. To our knowledge, there are no published randomized controlled trials evaluating the efficacy of therapeutic exercise in PFBT survivors. Results from small uncontrolled trials suggest that therapeutic exercise can positively impact a range of functional outcomes, including cardiorespiratory fitness, strength and bilateral coordination [29–32]. Furthermore, therapeutic exercise is associated with positive changes in brain architecture, which has been linked to improvements in motor planning, cognitive function and behavioral outcomes [33, 34]. Exercise interventions are also reported to enhance quality of life and reduce fatigue in childhood cancer survivors, but this has not been rigorously examined in PFBT survivors [28, 35, 36].

Goal-directed therapy aims to promote functional performance and independence in everyday activities, where goals are set by the patients and their families [37, 38]. An individualized, patient-centered approach not only promotes attention and motivation in therapy, but also provides a platform for delivering fully personalized therapeutic exercise programs for patients with significant impairments, activity limitations, and participation restrictions [39]. Goal-directed therapy aligns with the principles of the International Classification of Functioning, Disability and Health (ICF) framework, with particular focus on the activity and participation domains [40]. There is evidence supporting the effectiveness of family-centered, goal-directed interventions to improve motor performance, habitual physical activity, and quality of life in other pediatric patient groups, including cerebral palsy, muscular dystrophy, and intellectual disability [37, 39, 41]. No studies, however, have evaluated the efficacy of a goal-directed exercise intervention in any pediatric brain tumor survivor cohort.

### Aims and hypotheses

The Physical ACTivity in Survivorship (PACTS) study aims to evaluate the effects of a 12-week goal-directed therapeutic exercise program on cardiorespiratory fitness and physical activity-related goal attainment in PFBT survivors. We hypothesize that, compared with usual care, children who receive the intervention will achieve significantly greater gains in cardiorespiratory fitness and goal attainment, immediately post-intervention, with improvements maintained at 6- and 12-month follow-ups.

Secondary aims are to evaluate the effects of the exercise program on cardiorespiratory endurance, high-level mobility skills, functional muscle strength, habitual physical activity, gait, balance, quality of life, fatigue, participation, perceived movement skill competence and parameters of body composition. We hypothesize that, compared with usual care, children who receive the intervention will achieve significantly greater gains in these secondary outcomes immediately post-intervention, with improvements maintained at 6- and 12-months follow-ups.

## Methods

### Study design

This study is a single-center randomized controlled trial. Participants will be randomized into the intervention arm (*n* = 24) or usual care (n = 24). The study will be conducted in accordance with the Standard Protocol Items: Recommendations for Interventional Trials (SPIRIT) guidelines [42]. See SPIRIT Checklist in Additional file 1. Assessments for all participants will be conducted at baseline (T1), immediately post-intervention (12-weeks post baseline) (T2), and at 6-month (T3) and 12-month (T4) follow-up. Each assessment involves: 2 days of testing at a clinic visit, assessor-administered or self-administered (age dependent) child questionnaire, self-administered parent questionnaire, and monitoring of physical activity by accelerometer for 7 days. An overview of the study design, the schedule for enrollment and study assessments is shown in Table 1.

**Table 1:**
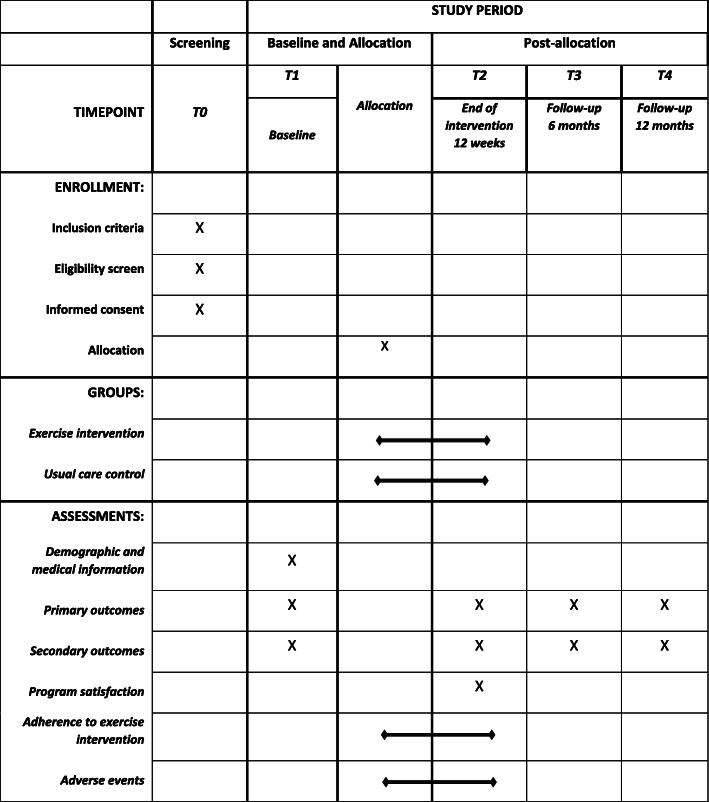
Schedule of enrollment, intervention, and assessment

Primary outcomes: cardiorespiratory fitness and physical activity-related goal attainment

Secondary outcomes: cardiorespiratory endurance, high-level mobility skills, functional muscle strength, habitual physical activity, gait, balance, quality of life, fatigue, participation, perceived movement skill competence and parameters of body composition

### Eligibility criteria

To be eligible participants must: be aged between five and 17 years; have had a diagnosis of a PFBT; undergone surgery for a PFBT a minimum of 12 months earlier, with no maximum time since surgery providing age eligibility is met; have completed radiation and/or chemotherapy in the previous 6 months; have no evidence of progressive disease; and, are deemed medically able to complete an exercise program, as determined by the treating oncologist. Participants will be excluded if: they have insufficient literacy skills to complete study assessments; or they are unable to travel to the research facility to complete study assessments or exercise intervention sessions.

### Recruitment

Potentially eligible participants will be identified through a patient database at the Pediatric Oncology Unit at Queensland Children’s Hospital, Brisbane, Australia. Potential participants may also be identified at weekly Pediatric Oncology multi-disciplinary meetings. Identified participants will be contacted by a Neuro-oncology Clinical Nurse via telephone, email, mail, or in person during their out-patient appointment, where they will be provided with a study information packet and eligibility screening will be completed. Following confirmation of eligibility and informed consent, a member of the research team will schedule the baseline assessment.

### Randomization, allocation, and blinding

The treatment allocation schedule will be generated and supervised by an independent statistician and concealed in Research Electronic Data Capture (REDCap). Using the randomization module in REDCap, participants will be randomized into the intervention or usual care group, stratified by treatment type (surgery only; surgery and radiation therapy; surgery, radiation therapy and chemotherapy). Within an exercise intervention, it is not possible to blind either therapist or participant; however, therapists will not take part in post-intervention outcome assessments. Trained assessors blinded to group allocation will collect outcome data.

### Therapeutic exercise intervention

Participants randomized to the intervention group will receive weekly individualised, goal-directed exercise therapy delivered face-to-face for 12 weeks, along with an accompanying home-based program. Each face-to-face session will be delivered by a qualified physiotherapist or accredited exercise physiologist (referred to hereafter as therapist), for a 30–60-min duration. The intervention will adhere to accepted exercise prescription guidelines for children and adolescents, comprising physical exercise training (cardiorespiratory and strength-promoting activities) and goal-directed task specific practice [43, 44]. At least half of the face-to-face session will be dedicated to cardiorespiratory-focused activities, with the remaining half dedicated to goal-directed activities. Goals selected will be physical activity- related and may include community recreation, sports, or leisure goals. The structure of the therapeutic exercise program, including the type, intensity and duration, will be individualized according to each participant’s age, goals, interests, functional capacity, presence of treatment-related side-effects and co-morbidities.

To supplement the face-to-face sessions, participants will complete a parent-led, home-based exercise program three times per week, up to 30 min of duration each time. The home-based program will replicate the essential elements of the supervised sessions in relation to activity type, intensity, and duration, and will also reflect the resources available at home. Prior to commencing face-to-face sessions, the therapist will complete a home visit to determine what space, equipment, and resources are available to complete the home-based exercise program safely and effectively. Parents will be given a detailed manual (including pictures and descriptions) for delivering the home-based component and will be encouraged to participate in the activities with their child. Peers and siblings may also be included to promote adherence to the home-based program. The home-based program will be monitored via heart rate monitors. The therapist will complete a follow-up home visit, between week four and week eight of the intervention, to provide an opportunity for participants and parents to ask questions about the home-based program, resolve any difficulties completing the program, and review the current activities.

#### Program fidelity

Due to the highly heterogenic nature of a goal-directed intervention, several strategies will be used to monitor compliance with the exercise program. First, the type, intensity and duration of each activity will be documented by the therapist using a standard form. The forms will prompt therapists to reflect on whether their session delivered the required dose of cardiorespiratory and goal-directed activities. Second, where possible, all sessions will be delivered by the same therapist to ensure consistency between sessions. Third, when consent is provided by parents or their guardian, intervention sessions will be video recorded and assessed by another member of the research team, with significant experience in pediatric exercise therapy. At the conclusion of the exercise intervention, parents will be asked to complete a questionnaire assessing their overall satisfaction with the program and whether the program met their expectations and needs.

### Usual care

Participants randomized to the control group will receive care as usual. Usual care is highly variable for children who are PFBT survivors; however, it is anticipated that most participants will not receive direct therapy services for the duration of the study. Usual care may include any therapy (e.g., physiotherapy, occupational therapy, speech and language therapy) and visits to their treating medical practitioner.

### Participant characteristics

Upon meeting eligibility criteria and providing written consent, participants will complete a demographic and medical information questionnaire, provided to the parent up to 2 weeks prior to their scheduled baseline assessment. The following medical information will also be extracted from patient medical records by trained staff: definitive date of diagnosis, tumor type and grade, intraventricular shunt placement, extent of surgery, site and dose of radiation therapy, agent and cumulative dose of chemotherapy, hormone therapy, dose and frequency of Botulinum Toxin A (BoNTA), and physical impairment (e.g., ataxic gait, hemiplegia).

At each assessment, the following anthropometric measures will be taken: height (Seca 264 digital stationary stadiometer, Seca, Germany), weight (Seca 703, column scales, Seca, Germany), and waist circumference measured using a stretch resistant tape. Information about each participant’s usual care during the study period will be collected through a semi-structured parent interview.

### Primary outcomes

#### Cardiorespiratory fitness

A continuous ramped exercise test for determination of maximal aerobic capacity will be conducted on an electronically braked pediatric cycle ergometer (Ergoselect 150, Ergoline, Bitz, Germany). Following a 2-min warm-up at an output of 10 watts, power output will increase at a rate of 10 W per minute and cycling cadence will be maintained at 75 rpm (rpm) [45]. The test will continue until pedal cadence drops below 60 rpm for 5 consecutive seconds, at which point power output will be reduced to 10 W for a 5-min recovery. Peak oxygen consumption (Peak *V*O_2_) will be determined using the methods described by Day et al. [46]. Breath-by-breath changes in gas exchange and ventilation will be measured using the MetaMax-3B portable metabolic analyzer (Cortex, Leipzig, Germany). Oxygen consumption (*V*O_2_) will be averaged over five second time windows. This measure has been shown to be reliable among children (ICC = 0.96) [47].

#### Goal attainment

The Canadian Occupational Performance Measure (COPM) is a semi-structured interview used to identify participant issues of personal importance in the domains of self-care, productivity and leisure [48]. Only goals pertaining to active recreation will be considered for this study. Children and parents will be asked to identify activities the child has difficulty performing, and to rate each activity on a scale of importance, rated from 1 (lowest) and 10 (highest). Up to five priority goals will be identified using the importance rating, and these are then rated on a scale of performance and satisfaction, using a similar 10-point scale [49]. The COPM will be administered during baseline assessment. The goals set by children allocated to the intervention group will be used to guide activity selection for the face-to-face intervention sessions and home-based program. Ratings from the parent for children under 8 years of age, and ratings from children over 8 years, in collaboration with their parent, will be reported [50, 51]. The COPM test-retest reliability is high (ICC 0.76–0.89) and is responsive to change in pediatric clinical trials [40, 52, 53]. A change of two points or greater has been reported as being clinically significant [48].

### Secondary outcomes

#### Cardiorespiratory endurance

The Modified Shuttle Test requires participants to walk/run back and forth along a 10-m course in time with a sound signal recording [54]. Speed for the first minute is set to 1.8 km/hour and increased by 0.61 km/hour every minute thereafter. The test is terminated when participants can no longer follow the pace set by the recording and the last lap successfully completed is recorded [55]. Heart rate and perceived effort using the OMNI scale will be recorded just prior to the test and immediately following test termination [56]. The Modified Shuttle Test has been used in pediatric patients with cystic fibrosis and has been shown to be a reproducible and valid measure of cardiorespiratory endurance [55, 57].

#### High-level mobility skills

High-Level Mobility Assessment Tool (HiMAT) is a 13-item tool assessing the performance of high-level mobility skills including walking, running, skipping, hopping, stairs, and bounding, among people who have sustained a brain injury [58]. Raw scores for all items will be recorded and scored on a scale between zero and four, where a score of zero represents the inability to perform an item, and a score of one to four represents increasing levels of ability. An additional five points for each stair item, will be awarded to the participant if they can ascend and/or descend the stairs using a reciprocal lower limb pattern and without the assistance of a rail. The HiMAT has demonstrated very high interrater reliability (0.99), retest reliability (0.98–0.99), and internal consistency (0.97) in patients with acquired brain injury, including children with tumors [58, 59]. Furthermore, the HiMAT did not exhibit a ceiling effect in children with an acquired brain injury [59].

#### Functional muscle strength

Functional muscle strength will be measured by identifying 30 s repetition maximum, preceded by a practice trial, for the following exercises: sit-to-stand, and half-kneel to stand, based on the protocol outlined in Verschuren et al. [60]. These assessments have evidence of reliability (ICC range: 0.84–0.98) in children with acquired brain injuries (including brain tumors) [59].

#### Habitual physical activity

Daily moderate-to-vigorous physical activity will be measured using the ActiGraph GT3X+ triaxial accelerometer (ActiGraph Corporation, Pensacola, FL, USA). Monitors will be worn 24 h per day on their non-dominant wrist for seven consecutive days. Upon return of the accelerometer, the raw accelerometer data will be downloaded and processed into physical activity metrics using a random forest physical activity classification algorithm for children [61, 62]. This validated algorithm uses patterns or features from the raw tri-axial acceleration signal to quantify daily time spent in sedentary activities (sitting or lying down), light-intensity activities and games, walking, running, and moderate-to-vigorous intensity activities and games. Non-wear time will be quantified by summing the prediction windows in which the standard deviation of the acceleration signal was < 13 mg for ≥30 consecutive minutes [63].

#### Gait

The GAITRite system (GAITRite Electronic Walkway, CIR Systems Inc.) will be used to measure real-time temporal and spatial parameters of gait, including velocity, cadence, step length, stride length, heel-to-heel base of support, single leg support (left and right), double leg support, and toe in/out angles [64]. Participants will walk at a self-selected comfortable walking speed across a pressure-sensor mat. The GAITRite system is a valid and reliable tool for measuring both averaged and individual step parameters of gait in children, with ICC ranging between 0.57 and 0.93 [64, 65].

#### Balance

Balance will be assessed using a PASCO force plate (PASCO PASport 22-axis force platform PS-2142). Participants will be assessed in three stances; bilateral, right unilateral and left unilateral, under two conditions; barefoot contact and a foam surface placed on the force plate. Displacement of center of pressure and mean sway velocity in mediolateral and anteroposterior directions will be measured [66, 67].

#### Peripheral neuropathy

A modified battery of the pediatric-modified Total Neuropathy Scale (peds-mTNS) will assess lower extremity neurological signs and symptoms, to evaluate their contribution to movement and balance findings. The peds-mTNS comprises three sets of questions on sensory symptoms, pain, motor function and autonomic function, as well as a five-part non-invasive neurological exam, including light touch sensation, vibration sensation, pin sensation, distal muscle strength and deep tendon reflexes [68]. The ped-mTNS demonstrated excellent internal consistency (> 0.9) in school-aged children with chemotherapy-induced peripheral neuropathy [68]. To minimize potential confounding factors of central nervous system signs, plantar (Babinski) and clonus reflexes will also be tested [69, 70].

#### Quality of life (QoL)

The Pediatric Quality of Life Inventory (PedsQL) Generic Core Scales 4.0 comprise 23 items measuring four dimensions of QoL; physical functioning, emotional functioning, social functioning and school functioning [71]. Each item utilizes a 5-point Likert response scale across child self-report and parent proxy-report (0 = never a problem to; 4 = almost always a problem), which will be reversed scored, with higher scores indicating better QoL. The measure consists of three age groups: 5 to 7 years (assessor-administered), eight to 12 years, and 13 to 17 years. The PedsQL will be completed by both parents and participants, with both child- and parent-report total scale scores reporting internal consistency of 0.90 [71]. This measure has demonstrated reliability and validity in samples of healthy children and pediatric patients with acute or chronic health conditions (including cancer) [72–74].

Parents and children will also complete the PedsQL Brain Tumor Module [74]. This disease-specific module includes 24 items encompassing six subscales; cognitive problems, pain and hurt, movement and balance, procedural anxiety, nausea and worry. To ensure all questions are considered age-appropriate, the child-report measure excludes the item “trouble writing school papers and reports”, for ages five to seven. The PedsQL Brain Tumor Module has demonstrated reliability and validity in a sample of children with brain tumors, with internal consistency of 0.78–0.92 for the parent-report score and 0.76–0.87 for child self-report score [73].

#### Fatigue

Pediatric Quality of Life Inventory Multidimensional Fatigue Scale (PedsQL-MFS) will be completed by parents and children, including 18 items encompassing three subscales; general fatigue, sleep/rest fatigue, and cognitive fatigue. The format, instructions and Likert response scale are identical to the PedsQL Generic Core Scales, with higher scores indicating less fatigue. PedsQL-MFS has demonstrated internal consistency, reliability and validity among a diverse sample of pediatric cancer patients (aged 2–18) [74]. Validity has been demonstrated in comparisons with healthy, non-cancer pediatric populations [74].

#### Participation

The Participation and Environment Measure for Children and Youth (PEM-CY) is a 20-item instrument completed by the parent and examines the frequency and level of participation across three settings; home, school and community [75]. For each item, parents will be asked to rate: (1) the frequency of their child’s participation in the stated activity on an 8-point scale (daily to never); (2) how involved their child typically is while participating on a 5-point scale (very involved to minimally involved); and (3) whether the parent would like to see a change in their child’s level of participation (yes/no). If yes, the parent will be asked what kind of change they would like to see on a 5-point scale (more involved to less involved). After each section, parents will be asked to indicate whether certain features of the environment make it easier or harder for their child to participate. This measure has demonstrated reliability and validity in a sample of children and youth with and without disabilities (aged 5–17 years), with acceptable internal consistency [75].

#### Perceived movement competence

The Perceived Movement Skill Competence for Young Children is a pictorial tool that scores a child’s perceived movement competence for fundamental movement skills [76]. It involves the participant looking at picture cards of fundamental movement skills that include six locomotor items (run, gallop, hop, leap, jump, glide) and six object control items (strike a ball, bounce a ball, catch, kick, overarm throw and underarm roll). Each card shows a picture of a ‘poor’ or ‘good’ performance of that skill and the participant will be asked to choose which picture they most identify with. In a sample of 23 children, internal consistency was acceptable (≥0.60) for both locomotor and object control [76]. Test-re-test reliability over a one-week period was excellent (≥0.83) [76].

#### Parameters of body composition

Total body scans using dual-energy X-ray absorptiometry (DXA; Prodigy, GE Medical Systems, LUNAR, Madison, WI, USA) will acquire parameters of bone mineral density (BMD, g/cm^2^) and body composition (total body lean and fat mass, kg) data. Scans of the anterior-posterior spines L1-L4, left and right proximal femur will additionally be acquired. All scans will be performed as per standard protocols in lying supine and analyzed using the manufacturers pediatric software (EnCore version 14.1). Total body lean mass will be calculated as fat-free mass minus bone mineral content. DXA measured parameters of body composition and bone mineral density demonstrate a coefficient of variation of less than 2% [77, 78].

### Sample size and power

To detect the clinically important differences of 5.5 mL/kg/min for Peak *V*O_2_ [79] and 2 points on the COPM [48] with 80% power, a sample of 20 participants per group is required (*n* = 40). This calculation assumes a two-tailed alpha level of 0.05, and standard deviations of 5.5 mL/kg/min and 1.5 points for peak *V*O_2_ [80] and the COPM score [81], respectively. To offset a projected attrition rate of up to 20%, we will recruit and enroll 24 participants per group, providing an overall sample size of 48.

### Statistical analysis

Analysis will follow standard principles for randomized controlled trials using two-group comparisons including all participants on an intention-to-treat basis. Between-group differences on the primary and secondary outcomes will be tested immediately post-intervention and at 6- and 12-month follow-up assessments using linear regression, with treatment group (exercise vs. usual care control) as the main effect and baseline measures of the respective outcome, along with other potential confounders (e.g., age, sex), as covariates.

### Ethics

Ethical approval has been granted from the human research ethics committee of Children’s Health Queensland (HREC/19/QCHQ/50464) and the Queensland University of Technology (1900000696). The trial has been prospectively registered with the Australian New Zealand Clinical Trials Registry (ACTRN12619000841178). Before enrolling in the trial, full written and informed consent will be obtained from legal parents/guardians of all participants, verbal assent for children aged seven to 13 years, and verbal and written assent for children aged 14 years or older. Participant data will be managed on a secured electronic database (REDCap) and hardcopy forms stored securely at the research facility.

### Safety and adverse events

A standardized protocol will be used to monitor adverse events that may occur during the face-to-face exercise sessions or home-based program. Adverse events commonly related to participating in physical activity may include, but are not limited to muscle soreness, falls, injury, exacerbation of existing symptoms, or any new or changed health problems since the previous contact. Adverse events will be graded using the Common Terminology Criteria for Adverse Events version 5.0 criteria [82]. Serious adverse events related to the intervention will be reported by the chief investigator to the lead ethics committee. Risk assessments with mitigation strategies will be implemented prior to commencing the home-based program.

## Discussion

This paper presents the rationale and design for a randomized controlled trial evaluating the efficacy of a 12-week goal-directed therapeutic exercise intervention in pediatric survivors of PFBTs. The increasing incidence and survival rates of PFBT indicate that there is a growing population of paediatric survivors of PFBT. However, there is a lack of research informing health care providers on effective interventions to manage the adverse effects of the disease and its treatment. To date, exercise interventions in childhood cancer survivors have implemented a structured exercise training approach, in group- and/or home-based settings. No studies have evaluated the efficacy of a goal-directed exercise intervention in any paediatric brain tumour survivor cohort, which may be associated with larger, more sustainable improvements physical functioning and habitual physical activity. Improvements in cardiorespiratory fitness and goal attainment may enhance the quality of survivorship and play a major role in mitigating the risk of disabling secondary health conditions later in life such as obesity, cardiovascular disease, osteoporosis, and metabolic disorders. The program of research will have strong potential for translation to clinical practice and pediatric rehabilitation services.

## Supplementary Information

**Additional file 1.** Standard Protocol Items: Recommendations for Intervention Trials (SPIRIT) Checklist”. The document contains a table listing the SPIRIT checklist criteria and the page number/s where each criterion is addressed in the manuscript.

## Data Availability

Not applicable.

## Abbreviations

BMD: Bone mineral density
COPM: Canadian Occupational Performance Measure
CTCAE: Common terminology criteria for adverse events
DXA: Dual-energy X-ray absorptiometry
HiMAT: High-level mobility assessment tool
ICF: International Classification for Functioning, Disability and Health
PACTS: Physical activity in survivorship
Ped-mTNS: Pediatric-Modified Total Neuropathy Scale
Peds-QL: Pediatric Quality of Life Inventory
Peds-QL MFS: Pediatric quality of life inventory multidimensional fatigue scale
PEM-CY: Participation and environment measure for children and youth
PFBT: Posterior fossa brain tumor
PMSC: Perceived movement competence skill scale
QoL: Quality of Life
QUT: Queensland University of Technology
VO_2_: Volume of oxygen consumed in one minute
VCO_2_: Volume of carbon dioxide expired in one minute
SPIRIT: Standard Protocol Items: Recommendations for Interventional Trials
